# Supplementary material for: EnzML: multi-label prediction of enzyme classes using InterPro signatures
Source: BMC Bioinformatics. 2012 Apr 25;13:61. doi: 10.1186/1471-2105-13-61 (PMC3483700; doi:10.1186/1471-2105-13-61)
Supplement: Addtional file 5 — The Java code to format the data files, evaluate and predict. The file enzml_java_code.tar.gz contains the Java code used to format database data to ARFF and XML formats, to execute cross and train-test (jackknife) evaluations and to record evaluation results to database. More information is included in the readme.txt file and the Javadoc files. The code can be used with a MySQL database. To use a different database software, other JDBC drivers might be required. [file 1471-2105-13-61-S5.gz › java_code/enzml2011/doc/uk/ac/ed/inf/enzml/mulan/attributesfilter/AttributesFilteredDataSetGenerator.html]

AttributesFilteredDataSetGenerator


---


|  |  |  |
| --- | --- | --- |
| **Overview** \| **Package** \| **Class** \| **Use** \| **Tree** \| **Deprecated** \| **Index** \| **Help** | |  |
| **PREV CLASS**   **NEXT CLASS** | **FRAMES**    **NO FRAMES**     **All Classes** |  |
| SUMMARY: NESTED \| FIELD \| CONSTR \| METHOD | DETAIL: FIELD \| CONSTR \| METHOD |  |


---


## uk.ac.ed.inf.enzml.mulan.attributesfilter Class AttributesFilteredDataSetGenerator

```
java.lang.Object
  uk.ac.ed.inf.enzml.weka.DataSetGenerator
      uk.ac.ed.inf.enzml.mulan.MulanDataSetGenerator
          uk.ac.ed.inf.enzml.mulan.attributesfilter.AttributesFilteredDataSetGenerator
```

---

``` public class AttributesFilteredDataSetGenerator extends MulanDataSetGenerator ```

Get passed a list of attributes, instead of taking it from
instances-attributes map.

**Version:**
:   3 Sep 2010

**Author:**
:   Luna De Ferrari luna.deferrari-at-ed.ac.uk

---

**Constructor Summary**

`AttributesFilteredDataSetGenerator(AttributesFilteredDataSetManager mulanDataSetManager,
uk.ac.ed.inf.utils.maputils.OneToManyMap<java.lang.String,java.lang.String> instanceAttributeMap,
uk.ac.ed.inf.utils.maputils.OneToManyMap<java.lang.String,java.lang.String> instanceClassMap,
java.util.TreeSet<java.lang.String> attributesNames)`


| **Method Summary** | |
| --- | --- |
| `AttributesFilteredDataSetManager` | `getManager()` |

**Methods inherited from class uk.ac.ed.inf.enzml.mulan.MulanDataSetGenerator**

`getNumberOfAttributes, getNumberOfInstanceAttributeCouples`

**Methods inherited from class uk.ac.ed.inf.enzml.weka.DataSetGenerator**

`fillAndWriteInstancesConditional, fillInstances, getAttributes, getAttributesFactory, getClassName, getInstanceAttributeMap, getInstanceClassMap, getInstances, getInstancesFactory, getInstancesNames, getNumberOfClassValues, getNumberOfInstanceClassCouples, getNumberOfInstances, setInstancesNames`

**Methods inherited from class java.lang.Object**

`equals, getClass, hashCode, notify, notifyAll, toString, wait, wait, wait`

**Constructor Detail**

### AttributesFilteredDataSetGenerator

```
public AttributesFilteredDataSetGenerator(AttributesFilteredDataSetManager mulanDataSetManager,
                                          uk.ac.ed.inf.utils.maputils.OneToManyMap<java.lang.String,java.lang.String> instanceAttributeMap,
                                          uk.ac.ed.inf.utils.maputils.OneToManyMap<java.lang.String,java.lang.String> instanceClassMap,
                                          java.util.TreeSet<java.lang.String> attributesNames)
```


**Method Detail**

### getManager

```
public AttributesFilteredDataSetManager getManager()
```

:   **Overrides:**: `getManager` in class `DataSetGenerator`


---


|  |  |  |
| --- | --- | --- |
| **Overview** \| **Package** \| **Class** \| **Use** \| **Tree** \| **Deprecated** \| **Index** \| **Help** | |  |
| **PREV CLASS**   **NEXT CLASS** | **FRAMES**    **NO FRAMES**     **All Classes** |  |
| SUMMARY: NESTED \| FIELD \| CONSTR \| METHOD | DETAIL: FIELD \| CONSTR \| METHOD |  |


---
